# Hsa_circ_0005230 is up-regulated and promotes gastric cancer cell invasion and migration via regulating the miR-1299/RHOT1 axis

**DOI:** 10.1080/21655979.2022.2036514

**Published:** 2022-02-16

**Authors:** Yan-Yu Peng, Dan Sun, Yan Xin

**Affiliations:** Laboratory of Gastrointestinal Onco-Pathology, Cancer Institute & General Surgery Institute, The First Affiliated Hospital of China Medical University, Shenyang, Liaoning Province, China

**Keywords:** Hsa_circ_0005230, miR-1299, RHOT1, gastric cancer, migration, invasion

## Abstract

Gastric cancer (GC) is one of the most common cancers in the world. Circular RNAs (circRNAs) are a class of non-coding RNAs that are widely expressed in eukaryotic cells. However, their role has been poorly understood in GC. This report aimed to explore the biological functions of hsa_circ_0005230 and its action mechanism in GC. This study validated that hsa_circ_0005230 was significantly up-regulated in 130 cases of GC tissues using qRT-PCR, and clinicopathological feature analysis revealed that its high expression was positively associated with histological grade, lymph node metastasis, TNM stages, and poor prognosis. In vitro, functional experiments showed that silencing hsa_circ_0005230 significantly decreased GC cell proliferation, invasion and migration capabilities. In addition, the major proteins of EMT (epithelial-mesenchymal transition) relevance have changed. In mechanism studies, bioinformatics analyses were used to predict the hsa_circ_0005230/miR-1299/RHOT1 axis and hsa_circ_0005230 may serve as a sponge for miR-1299 and indirectly regulate the expression of RHOT1. The regulated relationships between the molecules on the axis were verified using qRT-PCR and correlation analysis. Dual-luciferase reporter gene assay has been used to verify the binding site between miR-1299 and RHOT1. WB (Western blotting) and IHC (Immunohistochemical) were used to verify that RHOT1 may play the role of oncoprotein and affect the biological behavior of GC. Overall, hsa_circ_0005230 could enhance the EMT phenotype by promoting RHOT1 expression through sponging miR-1299, thus affecting the biological behavior of GC. Hsa_circ_0005230 can be easily identified as a potential diagnostic biomarker and assessment prognosis target for GC.

## Graphical Abstract

**Figure:**
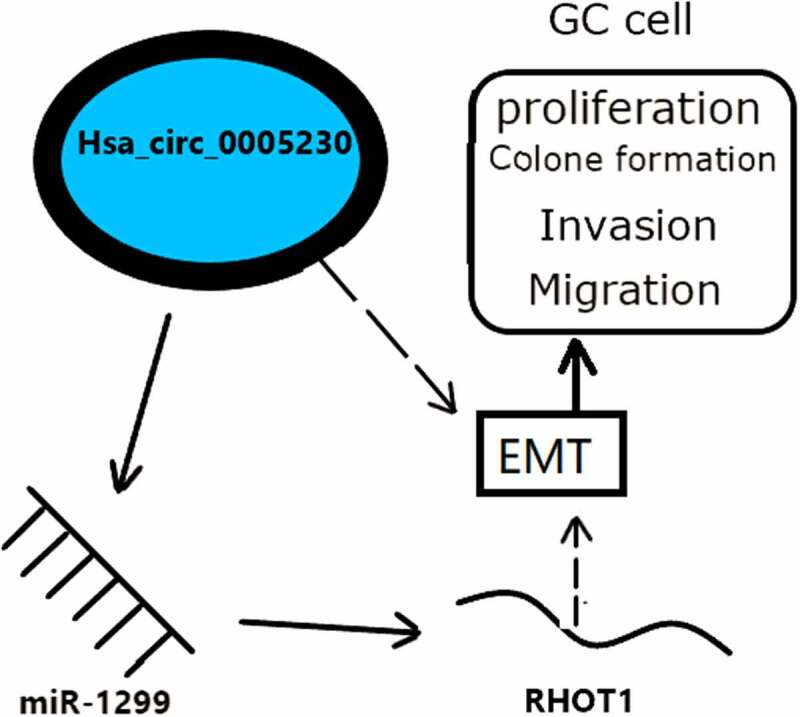

## Introduction

Gastric cancer (GC) is known as the common digestive system cancer with high morbidity and mortality rate in the world. According to the Global Cancer Statistics reported through assessing the incident and mortality rates of 36 types of cancer from 185 countries, GC was still the fifth most common cancer and the mortality dropped to fourth place from cancer [1]. Despite the positive use of early-stage diagnosis and radical surgery, the GC patients related five-year overall survival rate remains lower due to the invasive nature of the tumor [2]. At present, upper gastrointestinal (GI) endoscopy and pathological examinations are still the gold standards for the diagnosis of GC [3]. However, upper GI endoscopy plays as an invasive technique that is not routinely available and helps to evaluate as a physical examination in most countries. Therefore, identifying the molecular mechanisms of GC remains the first thing and it helps to determine the new diagnostic target markers and effective treatments.

CircularRNAs (circRNAs) have been discovered and confirmed in recent years as members of a family of functional non-coding RNAs with covalent closed-loop structures. Because of the resistance of circRNAs to RNase R digestion and its stable loop structure which is not easily broken down, circRNAs can exist stably in the cell and play its role. Aberrant expressions of circRNAs have been found such as in lung, breast, stomach, liver, and colorectal cancers. Furthermore, increasing pieces of evidence confirm that circRNAs have participated in the process of cancer including differentiation, proliferation, invasion, and migration. For instance, CircFBXL5 could sponge miR-660 to promote the progression of breast cancer [4]. Hsa_circ_0128846 was found to sponge miR-1184 and release AJUBA regulating the Hippo/YAP signal pathway affected tumorigenesis of colorectal cancer [5]. Hsa_circ_0000467 served as a diagnostic and prognostic biomarker for gastric cancer [6]. However, there are only a few circRNAs reported as GC biomarkers and currently, only a few of them have been associated with the invasion and migration of GC.

MicroRNAs (miRNAs), a class of post-transcriptional regulators, are involved in diverse cellular processes of cancers by interacting with the 3’-untranslated regions (3’-UTRs) of target genes. The disorders of miRNAs have been correlated with the occurrence and development of various cancers [7]. Ras homolog family (Rho) GTPases have been verified involving proliferation and invasion in various cancers. For instance, its family members like RhoA, RhoB, and RhoC have the potential to be activated by specific stimuli interacting with the different downstream effectors that play key roles in the distinct capacity of invasion and migration for cancer cells [8]. The competitive endogenous RNA (ceRNA) hypothesis proposed that RNAs sharing miRNA response elements (MREs) in their 3’-UTRs could impact the expression of miRNAs and induce gene silencing. The complex crosstalks of ceRNAs have been found in many different cancer types [9,10]. Numerous circRNA/miRNA/mRNA networks have now been predicted by bioinformatics analysis, and some have been shown to explain the mechanism of cancer [11,12]. Though little was known about circRNAs that can sponge miRNAs to influence GC, it was essential to determine circRNAs that have the potential to become GC biomarkers for diagnosis and assessment prognosis and to explore their mechanism in GC.

From our previous GC tissue microarray study, hsa_circ_0005230 was one of the circRNAs that was highly expressed in GC. The specific spliced of hsa_circ_0005230 is from gene DNM3OS (DNM3 opposite strand/antisense RNA), and located at chr1:172,109,619–172,113,577. It has also been reported to exert the effect of an oncogene in breast and cholangiocarcinoma [13,14]. Nevertheless, the essential effects by which hsa_circ_0005230 on GC are still unclear. Therefore, we selected hsa_circ_0005230 as a target molecule to research profoundly. The new member Ras homolog family member T1 (RHOT1), which had been studied in areas of mitochondrial transport and docking sites for mitophagy [15], lymphocyte migration [16], and so on. Previous studies indicated that RHOT1 could participate in cell invasion and migration of some tumors [16,17]. Nevertheless, RHOT1 in GC has not been explored and studies will be required. According to previous research results in other cancer, we chose the RHOT as the downstream target molecule of the axis.

Here, we hypothesized that hsa_circ_0005230 could sponge miR-1299, and RHOT1 could be used as a target gene of miR-1299. Hsa_circ_0005230 affected the expression level of RHOT1 in GC by sponge adsorption of miR-1299. This study aimed to investigate the role of hsa_circ_0005230 in GC and to explore the mechanism by which it influenced the biological behavior of GC. Therefore, this study might provide new evidence for hsa_circ_0005230 as a therapy target for GC.

## Materials and methods

### Clinical specimens and ethical statement

A total of 130 cases of fresh tissue samples of GC and paired noncancerous tissue samples were obtained from the First Affiliated Hospital of China Medical University between 2018 and 2020 (Ethics Review [2018] No. 88). Regarding normal gastric mucosal tissue, it was defined as at least 5 cm away from the primary cancer border and further confirmed by an experienced pathologist. These tissue specimens were promptly dropped into liquid nitrogen for 30 min and stored in a − 80°C refrigerator. All enrolled patients did not receive chemotherapy or radiotherapy before surgery. The research was approved by the Ethics Committee of the First Affiliated Hospital of China Medical University and the educated consensus was obtained from the patients before sample collection.

### Total RNA extraction and quantitative real-time PCR (qRT-PCR)

As recommended by the protocol, under RNase-free conditions, the miRcute miRNA kit (Tiangen, CHINA) was applied to extract total RNA. All qualified mRNA and circRNA were reverse transcribed by applying the PrimeScirpt RT Master Mix kit (Takara, China), while miRNA using the MIR-X miRNA First-Strand Synthesis Kit (Tiangen, CHINA) for reverse transcription. SYBR Green Master Mix Kit (Monad, CHINA) was applied to detect the relative expression of RNA. The specific primers were shown in Table 1. Reverse primer for miRNA was the universal reverse primer provided by the reverse transcription kit. Hsa_circ_0005230 and RHOT1expression levels were standardized using GAPDH and miR-1299 expression levels were standardized using U6. Each sample was run in triplicate.

**Table 1.** Specific primers for qRT-PCR

Primer name	Primer sequence (5′-3′)	Length (bp)
hsa_circ_0005230	F: 5’-CTCTTTGTTTTGCACACTAGGGA-3’	23
	R: 5’-ACCAGGTGAGCAGTCAAGAA-3’	20
miR-1299	F: 5’-TTCTGGAATTCTGTGTGAGGGA-3’	22
U6	F: 5’-TTCTGGAATTCTGTGTGAGGGA-3’	22
	R: 5’-GGAACGATACAGACAACATTAGC-3’	23
RHOT1	F: 5’-CTGATTTCTGCAGGAAACACAA-3’	22
	R: 5’-GCAAAAACAGTAGCACCAAAAC-3’	22
GAPDH	F: 5’-GAGTCAACGGATTTGGTCGT-3’	20
	R: 5’- TTGATTTTGGAGGGATCTCG-3’	20

### Cells culture and siRNA transfection

GES-1 and GC cell lines SGC-7901, BGC-823, AGS, HGC-27, and MKN-45 were conserved in the Gastrointestinal Tumor Pathology Laboratory of China Medical University. The above cells were added to RPMI-1640 medium (Gibco, NY, USA) with 10% fetal bovine serum cultured at 37°C in 5% CO_2_ with humidified air.

The two specific siRNAs si-circ-0005230-1/-2 derived from hsa_circ_0005230 and the negative control (NC) were processed by GenePharma (Shanghai, China). The sequences of si-circ-0005230-1/-2 were acquired from previous research references [14] and the sequences of siRNA were shown in Supplementary Table 1. Transfection of siRNAs in AGS and HGC-27 cells seeded in six-well plates. Transfected cells with 20 µM siRNA with Lipofectamine 3000 (Invitrogen Carlsbad, USA) transfection reagent. 48 hours later, the efficiency of silence was detected and determined by qRT-PCR.

### Clone formation

Clone formation assay for evaluating the capacity of transfected cells for cloning [18]. Cells were traditionally digested and cell concentration was adapted after logarithmic digestion. Two hundred cells were injected into each well of a six-well plate. Triplicates of each set of cells were made and cultured at 5% CO_2_ and 37°C. After visual inspection of the formed cell line clones, washed by phosphate-buffered saline (PBS) three times, immobilized for 15 min with 4% paraformaldehyde, then with Giemsa stain for 15 min, and discarded the staining solution before washing with PBS. Finally, the cloned cells (>50 cells) were counted visually and photographed by a digital camera. All experiments were performed at least three times.

### CCK-8 proliferation assay

In this experiment, the proliferation of AGS and HGC-27 cells was tested by CCK-8 kit (Beyotime Biotechnology, Shanghai, China) which was conducted as previously described [19]. Proceed in triplicate by incubating roughly 2 × 10^3^ cells in 100 μL in a 96-well plate at 37°C. The CCK-8 reagent (10 μL) was incorporated into each well at 0, 24, 48, 72, and 96 hours, and the incubation was carried out at 37°C for 2 hours. A microplate reader quantified OD values at 450 nM(Bio-Rad, USA). The results were analyzed using the average of the three measurements. Line charts were conducted using GraphPad Prism 5.0 software (La Jolla, USA).

### Transwell assay

Transwell experiments evaluated cell capacities of invasion and migration [20]. In the invasion experiment, Matrigel gel (BD, Franklin Lakes, NJ, USA) with serum-free RPMI 1640 medium = 1:7 dilution. The 50 μL of diluted Matrigel gel would be inserted into the transwell and incubated at 37°C for 2 hours. In the migration experiment, 200 μL of cell suspension was incorporated in a Transwell (Corning Inc., Corning, NY, USA) top chamber and 500 μL of RPMI medium including 20% fetal bovine serum was incorporated in a Transwell bottom chamber and the incubation was processed with 5% CO_2_ in a 37°C incubator for 48 hours. These chambers for the above experiments would be cleaned with PBS three times and fixed for 30 min with 4% paraformaldehyde, then stained for 15 min with 0.1% crystal violet. The counts of migrating and invading cell numbers were performed under an inversion microscope, and the average cell count of three fields was taken.

### Flow cytometry

We used a flow cytometry assay to detect the cell cycle [21]. The transfected GC cells have been stained with propidium iodide (PI, Beyotime, Beijing, China). Cells were collected from each group of logarithmic growth stages in EP tubes, and using PBS to wash three times, and then stored at 4°C overnight after fixed pre-chilled 70% alcohol. Treated cells were stained at 37°C for 30 min with PI. Flow cytometry was used to analyze using a FACSCanto II flow cytometer (FACSCalibur BD, USA). Experiments were repeated 3 times and data were conducted using the ModFit LT software (Verity Software House, Topsham, ME).

### Scratch wound assay

Scratch wound assay was used to assess the invasion and migration capabilities of GC cells [22]. Trypsin digested groups of cells and adjusted cell concentration to 3x10^5^/ml. Injection of 1 ml of cell suspension into six-well plate overnight incubation, using 10 μL sterilization gun to scrap the cell-free area in culture wells evenly, PBS rinsed 2–3 times, adding 2% serum medium 0 h, 24 h, and 48 h after the addition of culture medium to record the scratch cell wound under the microscope to take photos. Scratch wound rate = (0 h scratch width – scratch width)/0 h scratch width x 100%.

### Luciferase reporter assay

Determination of luciferase activity using a Dual-luciferase Reporter assay kit (GeneChem, Shanghai, China) according to the manufacturer’s protocol. The RHOT1 wild type and mutant type over-expression plasmids were acquired from Genechem (Shanghai, China). The 293T cell was seeded in 96-well plates, under 5% CO_2_ and 10% FBS cultured at 37°C. After co-transfection of luciferase reporter plasmid and miR-1299 up-expression plasmids or NC plasmids into293T cells for 48 hours, luciferase activity was assessed. RHOT1–3ʹUTR Wt reporter plasmid and RHOT1–3ʹUTR Mut reporter plasmid were constructed with the pmirGLO-promoter vector. Then detected the luciferase activity of fireflies and Renilla. The experiments were performed in triplicate.

### Immunohistochemical (IHC) staining

The protein expression of RHOT1 in GC tissues was analyzed by immunohistochemistry staining. Immunochemical staining treatments were performed as described previously, following standard protocols [23]. The paraffin sections of GC tissue samples and noncancerous tissue were dehydrated using the graded ethanol and incubated. Incubate slides with primary antibody (an anti-rabbit polyclonal antibody against RHOT1, 1:50 Biorbyt, England) at 4°C overnight. Then, cleaned with PBS buffer and stained with secondary antibody (goat anti‐rabbit, ZSGB-BIO, China). Images were taken with an inverted microscope (Nikon Corporation, Tokyo, Japan). Two high-power fields were selected for each case, and approximately 200 cells were counted, and the grade was determined based on the proportion of staining-positive cells among the same type of cells. The grade standards for staining of RHOT1 protein in tissues were as follows: 0, no cytosolic or cytoplasmic staining; 1, 5–25% of cells had cytosolic or cytoplasmic staining; 2, 26–50% of cells had cytosolic or cytoplasmic staining; 3, 51–75% of cells had significant cytosolic or cytoplasmic staining; and 4, 26–50% of cells had cytosolic or cytoplasmic staining. The distribution of intensity was defined as 0 = no staining, 1 = weak, 2 = moderate, 3 = strong, 4 = significantly strong. The intensity score grade was commonly determined on the product of the staining intensity and the percentage of cells as the intensity score grade. The intensity score classes were as follows: 0 = no staining (negative), 1–4 = weak staining (+), 5–8 = moderate staining (++), 9–12 = strong staining (+++). The results were assessed by two pathologists according to staining intensity and staining distribution.

### Western blotting(WB)

To ascertain the protein expression of RHOT1, the WB assay was carried out [22]. The collected cells were lysed using RIPA buffer containing PMSF at 4°C, then centrifuged and the protein quantification was performed by BCA Protein Quantification Kit (ComWin Biotech, Beijing, China) and boiled to denature at 99°C for 5 min. Separated proteins by 10% SDS-PAGE gel electrophoresis, with semi-dry transfer to PVDF membranes, then using 5% skimmed milk blocked for 1 h. The membrane was washed with TBST after blocking. Primary antibodies including RHOT1 (Biorbyt, UK, 1:750) E-cadherin (1:1000), N-cadherin (1:1000), Vimentin (1:1000), Snail (1:1000) and GAPDH (ZSGB-BIO, China, 1:1000) were added and incubated overnight on a shaker at 4°C. The secondary antibody (goat anti-rabbit) (Proteintech, USA, 1:5000) was then incubated for 2 hours. The protein band signals were analyzed using enhanced chemiluminescence reagents (ECL, Millipore, Burlington, MA, USA). The results were treated by Image J 1.8.0 software (National Institutes of Health, Bethesda, MD, USA) to calculate the grayscale values of the bands for subsequent analysis.

### Statistical analysis

The mean ± SD was used to express all experimental data results and analyzed using SPSS version 22.0 software (IBM, Armonk, NY, United States) and GraphPad Prism 5.0 (GraphPad Software, La Jolla, CA, United States). The expression of genes was shown using the 2^−ΔΔCT^method. The results used the Student’s *t*-test to analyze the significant difference. The Spearman rank correlation test was deployed to quantify a possible correlation. The differences between molecular expression and clinical characteristics of GC were tested by *χ*^2^ test. *P* < 0.05 was expected to be statistically significant.

## Results

We validated the role of hsa_circ_0005230 in GC. We examined the expression of hsa_circ_0005230 in GC and performed cell proliferation, invasion and migration using si-circ_0005230 to assess its biological behavior. The expression and function of downstream miR-1299/RHOT1 of the axis in GC were predicted and detected by bioinformatics. We found that silencing of hsa_circ_0005230 inhibited the proliferation, invasion and migration of GC cells. Thus, we provided new evidence for hsa_circ_0005230 as a treatment target for GC.

### Hsa_circ_0005230 is up-regulated in gastric cancer

**Figure 1.**
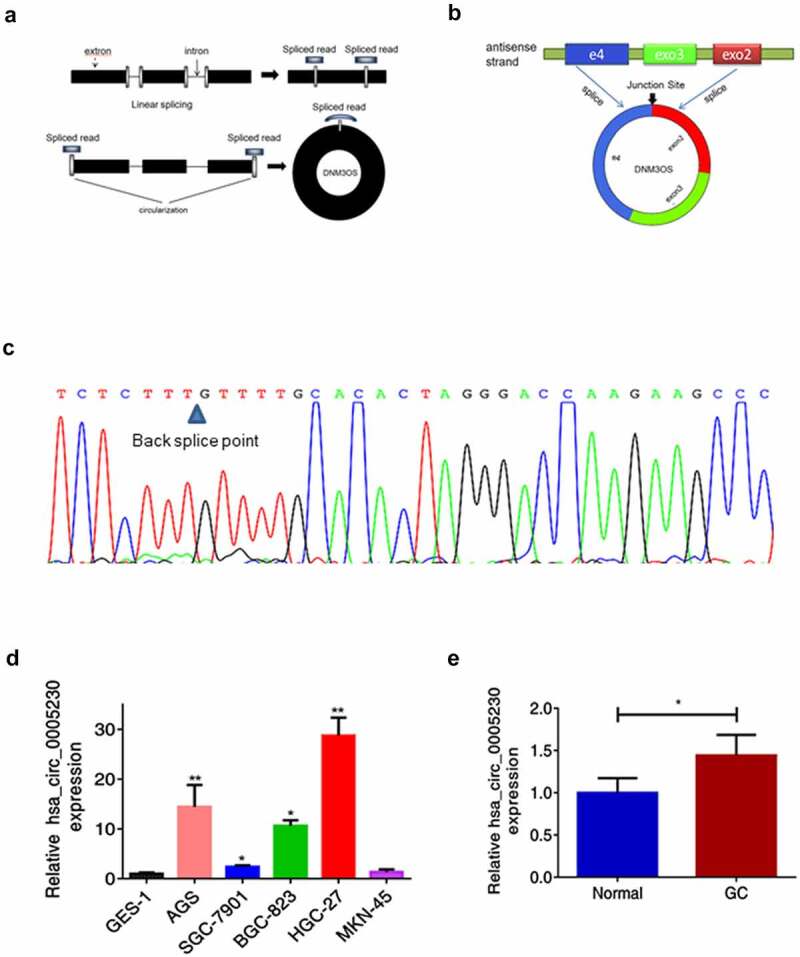
The biological structure of hsa_circ_0005230 and its expression in GC tissues and cells.

Using the website circBase (http://www.circbase.org/) acquired the hsa_circ_0005230 basic information. It was formed from linear DNM3OS (Figure 1 a). The software circPrimer (version 1.2.0.5) showed that hsa_circ_0005230 consisted of DNM3OS exon 2, 3, and part of exon 4(Figure 1b). Correct primers with the back-splice formation and amplified PCR products were tested by Sanger sequencing (Figure 1c). To identify the functional role of hsa_circ_0005230 in GC, firstly, the application of qRT-PCR analysis was performed to show expression in cells and tissues. The results revealed that compared with the GES-1 cell line, the expression of hsa_circ_0005230 was up-regulated in SGC-7901, BGC-823, AGS and HGC-27 (Figure 1d) and compared with paired noncancerous tissues, it was also up-regulated in 130 cases of GC tissues (Figure 1e). Second, we further calculated the correlation among hsa_circ_0005230 expressions and varied clinicopathological features of GC patients to determine the hsa_circ_0005230ʹs potential biomarker possibility in GC. Based on the expression of hsa_circ_0005230 of GC tissues from PCR assay results, the enrolled 130 GC patients were classified into two groups (the hsa_circ_0005230 expression of GC/Normal >1 defined as high expression group, and the surplus defined as low expression group). The results demonstrated that the high expression of hsa_circ_0005230 showed a positive correlation with histological grade (*P* = 0.041), lymph node metastasis (*P* = 0.021), and TNM staging (*P* = 0.006). However, the other clinicopathological features of GC such as gender, age, location, WHO’s histological types, Gross types, Lauren’s types, depth of invasion, and distant metastasis were not correlated with the expression of hsa_circ_0005230 (Table 2).

**Table 2.** Relationship between different hsa_circ_0005230 expression and clinicopathological features of GC

Clinic characteristics	Total	hsa_circ_0005230		χ^2^	*P*-value
		High(%)	Low		
Gender				2.148	0.143
Male	93	63 (67.7)	30		
Female	37	20 (54.1)	17		
Age(year)				0.129	0.72
≤60	39	24 (61.5)	15		
>60	91	59 (64.8)	32		
Location					0.104
Gastroesophageal	8	6 (75)	2		
Fundus/Cardia	5	5 (100)	0		
body	34	19 (55.9)	15		
Antrum	81	51 (63)	30		
Total stomach	2	2 (100)	0		
Tumor size(cm)				0.127	0.722
≤5	58	38 (65.5)	20		
>5	72	45 (62.5)	27		
Gross types					
EGC					
I	1	0 (0)	1		
III	2	2 (100)	0		
AGC					0.055
Bor.I+ II	4	4 (100)	0		
Bor.III+IV	123	77 (62.6)	46		
WHO’s histological types					0.155
Papillary adenocarcinoma	5	5 (100)	0		
Tubular adenocarcinoma					
Well differentiated	1	1()	0		
Moderately differentiated	34	22 (64.7)	12		
Poorly differentiated adenocarcinoma	63	38 (60.3)	25		
Undifferentiated carcinoma	1	1 (100)	0		
Signet ring cell carcinoma	13	10 (76.9)	3		
Mucinous adenocarcinoma	13	6 (46.2)	7		
Histological grade					0.041*
G1+ G2	36	28 (77.8)	8		
G3	94	55 (58.5)	39		
Lauren’s types				1.757	0.415
Intestinal	25	18 (72.0)	7		
Diffuse	76	45 (59.2)	31		
Mixed	29	20 (69)	9		
Depth of invasion(T)					0.662
T1+ T2	7	5 (71.4)	2		
T3+ T4	123	78 (63.4)	45		
Lymph node metastasis (N)				7.754	0.021*
N0	31	16 (51.6)	15		
N1	23	11 (47.8)	12		
N2-3	76	56 (73.7)	20		
Distant metastasis (M)					0.614
M0	127	82 (64.6)	45		
M1	3	1 (33.3)	2		
TNM staging				7.486	0.006*
I+ II	44	21 (47.7)	23		
III+IV	86	62 (72.1)	24		

Note: *P < 0.05.

Abbreviation: TNM, tumor–node–metastasis.

EGC, early gastric carcinoma.

AGC, advanced gastric carcinoma.

(a) Schematic diagram showed that according to the junction from back-splice of hsa_circ_0005230 designed the divergent primers. (b) Hsa_circ_0005230 was derived from DNM3OS exon 2, 3, and part of 4. (c) Sanger sequenceverified PCR amplification products of hsa_circ_0005230 were correct. (d) The expression of hsa_circ_0005230 was up-regulated in GC cell lines, compared with GES-1. (e) Hsa_circ_0005230 was up-regulated expression in 130 cases of GC tissues using qRT-PCR. The data were expressed as mean ± SD; *P < 0.05.

### Silencing hsa_circ_0005230 fairly inhibits the gastric cancer cells growth proliferation and arrests cells cycle in vitro

**Figure 2.**
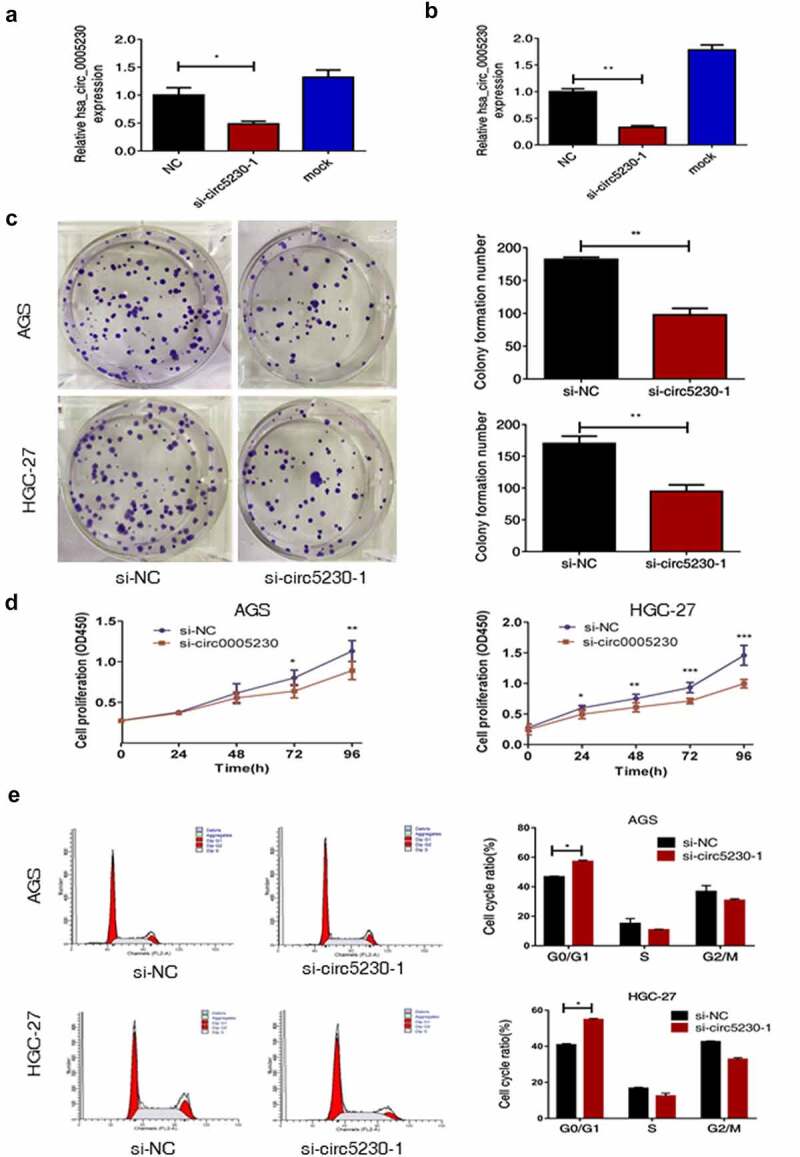
Silencing hsa_circ_0005230 not only diminished the capacities of clone formation and proliferation of GC cells but also arrested the cell cycle.

To deeply study the functional hsa_circ_0005230 in GC, we used specific siRNA silencing of hsa_circ_0005230 in AGS and HGC-27 cells which were the highest hsa_circ_0005230 expression. Firstly, qRT-PCR was used to detect the silencing efficiency of si-circ-0005230-1 and si-circ-0005230-2 in HGC-27 cell lines by transfected transiently with different concentrations, and the silencing efficiency greater than 50% was defined as effective. The results suggested that the silencing efficiency of si-circ-0005230-1 was better than si-circ-0005230-2 with transfected 6.25 μL (Supplementary Figure A). Subsequently, the silencing efficiency of si-circ-0005230-1 was examined in AGS (Figure 2a) and HGC-27 cells (Figure 2b), respectively, using si-NC as a control. Above the results indicated that si-circ-0005230-1 could successfully be silencing hsa_circ_0005230 for subsequent functional studies. Then, clone formation and CCK-8 assay were used to validate clonal forming and proliferation capability in AGS and HGC-27 cells. The results of the clone formation assay showed that silencing hsa_circ_0005230 expression significantly inhibited the number of colony cells and reduced the clonal forming capability in AGS and HGC-27 cells (Figure 2c). The CCK-8 assay results showed that the proliferation capacity of the si-circ-0005230 group was fairly inhibited in AGS cell (*P* < 0.05), after transfection 72 h and 96 h time, especially at 96 h (*P* < 0.01), compared with the si-NC group; and there were no fair differences between si-circ-0005230 group and si-NC group at the 24 h and 48 h time points in AGS cell. The changes in cell proliferation capacity were detected at the same observation time point in HGC-27 cells after silencing hsa_circ_0005230. The results showed that cell proliferation in the si-circ-0005230 group was inhibited at 24 h, 48 h, 72 h, and 96 h (*P* < 0.05) in HGC-27, especially at 48 h, 72 h, and 96 h (*P* < 0.01) (Figure 2d), compared with the si-NC group. The above indicated that the proliferation ability of GC cells was diminished after silencing hsa_circ_0005230 in AGS and HGC-27 cells. Furthermore, a flow cytometry assay was used to validate the distribution and changes in the GC cell cycle after silencing hsa_circ_0005230. The results indicated that the cell cycle proportion of G0/G1 phase in the si-circ-0005230 group was increased significantly both in AGS and HGC-27 cells (*P* < 0.05), as the proportion of S phase and G2/M phase, were reduced (*P* < 0.05), compared with the si-NC group (Figure 2e). It can be shown that silencing hsa_circ_0005230 significantly arrested the G0/G1 phase of cell cycle in GC cells, affecting the protein synthesis of cells in the S phase and reducing the proportion of S phase and G2/M phase, which was not advantageous to the mitotic phase of cells, thus affecting the cell division and proliferation activity.

(a) Utilizing qRT-PCR assay, compared with the NC group, si-circ-0005230-1 was effectively silenced in AGS. (b) Utilizing qRT-PCR assay, compared with the NC group, si-circ-0005230-1 was effectively silenced in HGC-27. (c) Clone formation assay observed that, compared with the NC group, silencing hsa_circ_0005230 significantly decreased the number of colonies in AGS and HGC-27 cells. (d) It was observed from the CCK-8 assay, compared with the NC group, silencing hsa_circ_0005230 declined proliferation of AGS and HGC-27 cells. (e) The use of flow cytometric analysis indicated that, compared with the NC group, silencing hsa_circ_0005230 arrested cell cycle progression in AGS and HGC-27 cells. The data were expressed as mean ± SD; *P < 0.05, **P < 0.01, ***P < 0.001.

### Silencing hsa_circ_0005230 predominantly suppresses the invasion and migration capacities of gastric cancer cells in vitro

**Figure 3.**
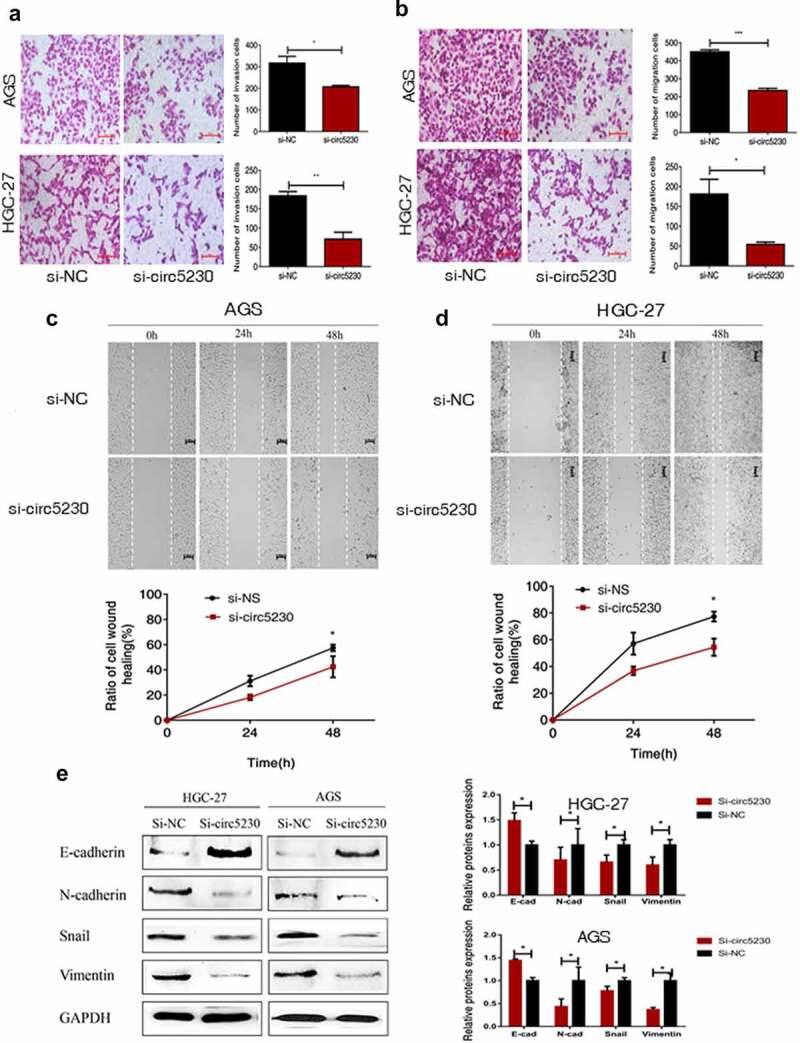
Silencing hsa_circ_0005230 inhibited the invasion and migration of GC cells.

We used transwell assay and scratch wound assay to explore the influence of hsa_circ_0005230 which was affecting the invasion and metastasis capacity of AGS and HGC-27 cells. First, the transwell invasion assay was executed. The results demonstrated the invasion capacity of GC cells was markedly reduced (Figure 3a). Then, a transwell migration assay was used to assess the effect of silencing hsa_circ_0005230 on the migratory capacity of AGS and HGC-27 cells. The results investigated that cell migration capacity was weakened in HGC-27 cell after silencing hsa_circ_0005230. The same results were exhibited in AGS cells (Figure 3b). The above mentioned investigated that silencing hsa_circ_0005230 could inhibit the potential invasion and metastasis capacity of GC cells. Furthermore, the scratch wound assay was also applied to estimate the capacity of cell migration after silencing hsa_circ_0005230 in AGS and HGC-27 cells. The results showed that the migration capacity of GC cells was weakened in the si-circ_0005230 group (Figures 3c, 3d); suggesting that silencing hsa_circ_0005230 could effectively reduce the migration capacity in AGS and HGC-27 GC cells. Following silencing of hsa_circ_0005230, the malignant biological behavior of GC cells such as proliferation, invasion, and migration was changed, considering that this may be related to the altered EMT (epithelial-mesenchymal transition) phenotype of GC. So we would detect the major proteins associated with the EMT phenotype. We detected the major mesenchymal phenotypic proteins, such as N-cadherin, Vimentin, and Snail, and the epithelial phenotypic protein E-cadherin by Western blot. The results indicated that after silencing hsa_circ_0005230, the expression of epithelial phenotype E-cadherin protein was enhanced, while that of the expression of mesenchymal phenotype N-cadherin, Vimentin, and Snail proteins diminished significantly both in AGS and HGC-27 cells (Figure 3e).

(a) The cell transwell invasion assay observed that silencing hsa_circ_0005230 expression decreased the potential capacity of GC cell invasion, compared with the NC group. (b) The cell transwell migration assay observed that silencing hsa_circ_0005230 expression inhibited the potential capacity of GC cell migration, compared with the NC group. (c) The scratch wound assay observed that silencing hsa_circ_0005230 expression inhibited the migratory capacity of AGS cells, compared with the NC group. (d) The scratch wound assay observed that silencing hsa_circ_0005230 expression inhibited the migratory capacity of HGC-27 cells, compared with the NC group. (e) Applying WB detected the changes of major protein expression of the EMT phenotype after silencing hsa_circ_0005230. The data were expressed as mean ± SD; *P < 0.05, **P < 0.01, ***P < 0.001.

### Hsa_circ_0005230 sponges miR-1299 in gastric cancer cell

**Figure 4.**
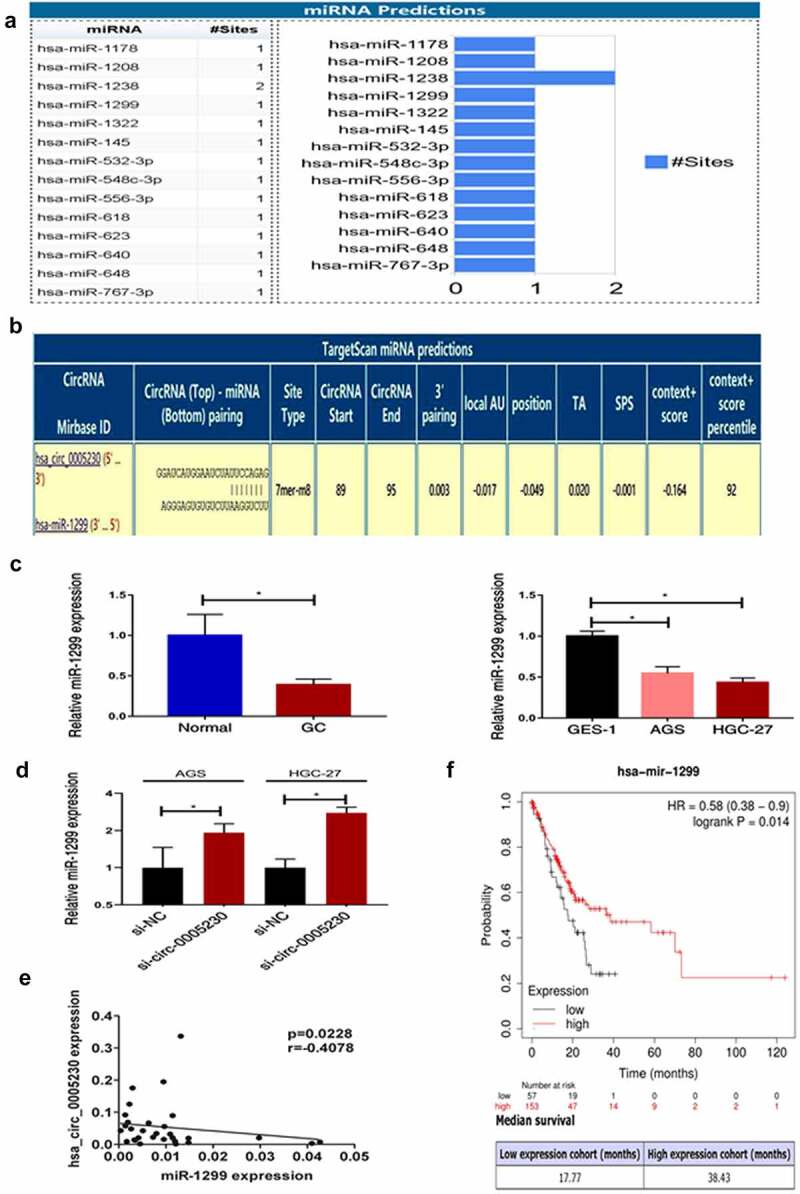
Hsa_circ_0005230 as a sponge to bind miR-1299.

Hsa_circ_0005230 as a differentially expressed functional RNA, to explore the molecular mechanism of hsa_circ_0005230 in regulating the biological behavior of GC cells is the next task. The localization of the circular RNAs in the cell determines the functions that they might perform. For example, a circular RNA localized in the cytoplasm may act as a ceRNA to compete for the regulation of downstream target genes. And circular RNAs localized in the nucleus may have the function of translating proteins or polypeptides [24]. According to hsa_circ_0005230 mainly located in the cytoplasm [13], we intended to use the mechanism of the ceRNA hypothesis explored circRNAs sponged miRNAs to indirectly regulate mRNA expression further influence the biological behavior of tumors. Searching Circinteractome (https://circinteractome.nia.nih.gov/index.html) there were 14 miRNAs revealed to have binding sites with hsa_circ_0005230 (Figure 4a). Previous research confirmed that miR-1299 played a suppressor role not only in various cancers but also in GC [25–28]. Targetscan (http://www.targetscan.org/vert_72/) displayed a predicted binding site between hsa_circ_0005230 and miR-1299 (Figure 4b), and hsa_circ_0005230 direct binding miR-1299 has been determined using the dual-luciferase reporter assay in the cholangiocarcinoma study [14]. Therefore, we intended to research miR-1299 deeply at tissue and cell level, then detected the expression of miR-1299 after silencing hsa_circ_0005230. The results demonstrated that at tissue level compared with paired noncancerous tissue, miR-1299 expression was fairly down-regulated in 33 cases of GC tissues. At cell level compared with GES-1, it was also fairly down-regulated in AGS and HGC-27 GC cells (Figure 4c). Subsequently, silencing hsa_circ_0005230 to detect miR-1299 expression was up-regulated in AGS and HGC-27 cells (Figure 4d). Furthermore, It has also been revealed that the negative correlation between hsa_circ_0005230 and miR-1299 analyzing the expression in GC tissues (Figure 4e). Besides, we performed a survival analysis of miR-1299 by using Kaplan–Meier Plot(http://www.kmplot.com/). The data of 210 cases of GC patients were included and divided into miR-1299 low and high expression groups, according to the best cutoff value(auto-selected). The results of survival analysis showed that when the mutation load was low, the overall survival in the miR-1299 high expression group (38.43 months) was significantly longer than the low expression group (17.77 months) (*P* = 0.014), and when the mutation load was high, there was no significant difference between the two groups (Figure 4f).

(a) It was predicted from the Circinteractome, 14 miRNAs have binding sites to hsa_circ_0005230. (b) The graph of Targetscan showed the predicted binding sites between miR-1299 and hsa_circ_0005230. (c) MiR-1299 expression was down-regulation in GC cells and 33 cases of GC tissues by qRT-PCR. (d) After silencing hsa_circ_0005230, it was detected miR-1299 was up-regulated expression in AGS and HGC-27 by qRT-PCR. (e) Hsa_circ_0005230 was negatively correlated with miR-1299 at the level of GC tissues. (f) Survival analysis from Kaplan-Meier indicates that survival is longer in GC patients with high miR-1299 expression. The data were expressed as mean ± SD; *P < 0.05, **P < 0.01.

### Hsa_circ_0005230 influences the expression of RHOT1 by sponging miR-1299

**Figure 5.**
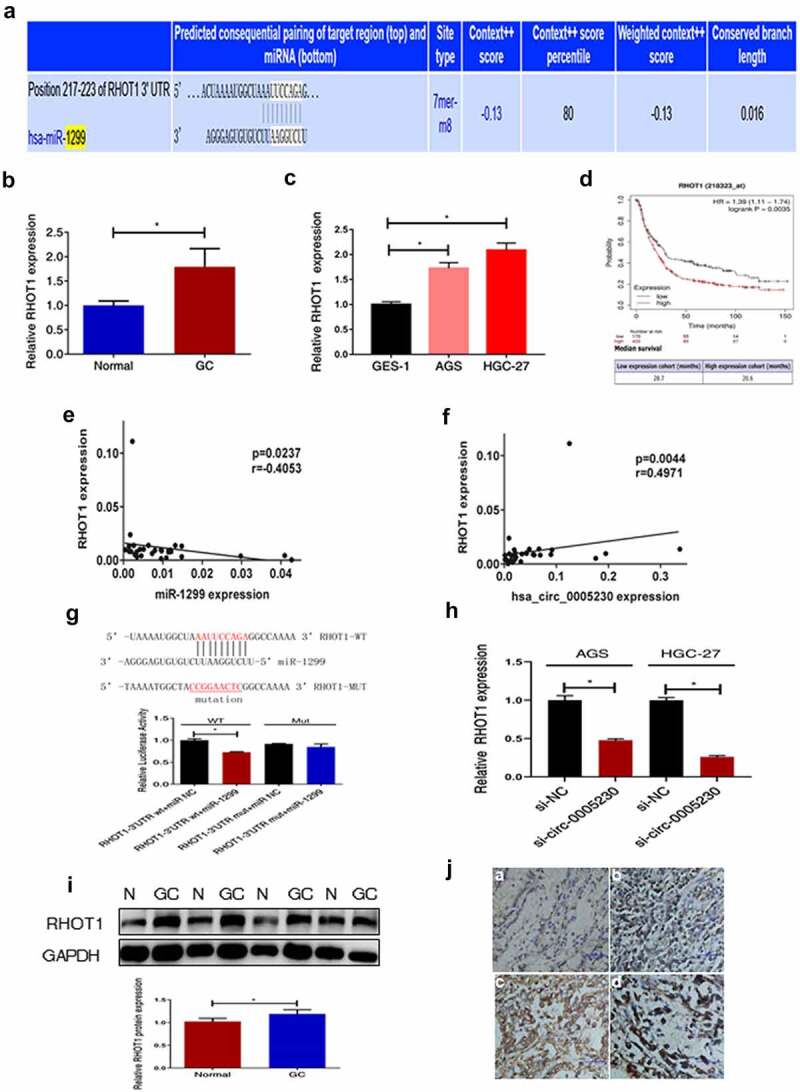
RHOT1 was the downstream target gene of the hsa_circ_0005230/miR-1299 axis.

As widely known, functional miRNAs were utilizing their specific sequences binding to the 3’-UTR of the downstream mRNA declined or inhibited translation of target mRNA. To further explore the downstream target genes of miR-1299, first, we used miRWalk (http://mirwalk.umm.uni-heidelberg.de/) to predict which downstream target mRNAs could bind with miR-1299. A lot of candidates have been predicted. From previous studies, we could know that RHOT1 as a new member of the GTPase family exerted a few functions in nervous system diseases and tumors. Though it acted as a positive role in the proliferation and invasion of pancreatic cancer [17], its function in GC was not clear. It was shown by miRWalk that miR-1299 had a binding site to RHOT1 as predicted (Figure 5a). Therefore, we selected RHOT1 as the target gene for our study, which may be regulated in expression level after hsa_circ_0005230 sponging miR-1299. Secondly, qRT-PCR was used to test the RHOT1 expression at tissue and cell levels. RHOT1 was up-regulated in 51 cases of GC tissues, compared with paired noncancerous tissues (Figure 5b), and compared with GES-1, it was also up-regulated in AGS and HGC-27 cells (Figure 5c). Afterward, we calculated the correlation between the level of RHOT1 expression and different clinicopathological features of GC patients. According to the relative expression of RHOT1 detected by PCR, when the expression of RHOT1 in the GC group was higher than that of RHOT1 in the normal group, it was defined as a high expression group; when the expression of RHOT1 in the GC group was lower than or equal to that of RHOT1 in the normal group, it was defined as a low expression group. The results demonstrated that the high expression of RHOT1 showed a positive correlation with lymph node metastasis (*P* = 0.035) and TNM staging (*P* = 0.042). However, the other clinicopathological features of GC such as gender, age, location, WHO’s histological types, Gross types, Lauren’s types, depth of invasion, and distant metastasis were not correlated with the expression level of RHOT1 (Supplementary Table 2). From the above results, it can be shown that the high expression of RHOT1 played a positive action to influence the invasion and migration of biological behavior of GC cells. Subsequently, we applied to assess the overall survival of RHOT1 using GSE29272 datasets from GEO in Kaplan-Meier Plotter. There were included 592 GC patients which were defined as groups of high and low expression, according to the best cutoff value 746 processed from the platform. We discovered that the high expression group of RHOT1 has short survival (Figure 5d). Consequently, patients with GC have a poor prognosis when RHOT1 was expressed at a high level. Afterward, correlation analysis with PCR data of GC tissues indicated that miR-1299 was negatively correlated with RHOT1 expression (Figure 5e), and equally crucial was that hsa_circ_0005230 expression was positively correlated with RHOT1 (figure 5f). Furthermore, the dual-luciferase reporter assay was conducted to verify the binding target relation between RHOT1 and miR-1299. The result was verified luciferase activity of RHOT1 3’-UTR-Wt was inhibited by transfection with miR-1299 up expression plasmids (*P* < 0.05), nevertheless the RHOT1 3’-UTR-Mut activity was not decreased (*P* > 0.05), suggesting that RHOT1 had specifically bound to miR-1299 (Figure 5g). To investigate whether after silencing hsa_circ_0005230 would interfere with the expression of the downstream target gene, we used qRT-PCR to detect the expression of RHOT1 when after transfected si-circ-0005230. The results showed that when after silencing hsa_circ_0005230, the RHOT1 expression diminished at the same time in AGS and HGC-27 cells (Figure 5h). Moreover, when completed above tests about RNA level of RHOT1, to investigate the expression level of RHOT1 protein, it was detected by WB and IHC staining in GC and paired normal mucosa tissues. The results indicated that RHOT1 was up-regulated in 48 cases of GC tissues, compared with paired normal mucosa tissues by WB assay (Figure 5i). And the expression level of RHOT1 was consistent both in RNA and protein levels in GC tissues. Using the IHC staining, we analyzed the pictures of155 cases of tissues of GC and 136 cases of normal mucosa after staining. The result indicated that RHOT1 protein was primarily localized in the cytoplasm. Analyzing from the staining pictures, the positive expression was predominantly in poorly differentiated GC tissues with or without Lymph node migration, also the poorer the histological differentiation or with lymph node metastasis, the stronger the RHOT1 expression (Supplementary Table 3) (Figure 5j). Furthermore, we calculated the correlation between the protein levels of RHOT1 in GC tissues and the clinicopathological features of GC at the same time. The staining expression levels of RHOT1 protein in 155 GC patients were classified into high expression group (score >1) and low expression group (score ≤1) according to staining intensity for statistical analysis of clinicopathological data. The results demonstrated that the high expression of RHOT1 showed a positive correlation with histological grade (*P* = 0.03), Lauren’s types (*P* = 0.012), depth of invasion (*P* = 0.038), lymph node metastasis (*P* = 0.015), TNM staging (*P* < 0.01). However, the other clinicopathological features of GC such as gender, age, location, WHO’s histological types, Lauren’s types, and distant metastasis were not correlated with the expression level of RHOT1 (Supplementary Table 4).

(a) As can be seen from the graph, miRWalk predicted that miR-1299 had a binding site with RHOT1. (b) RHOT1 was up-regulated in 51 cases of GC tissues by qRT-PCR. (c RHOT1 was up-regulated in GC cells by qRT-PCR. (d) An analysis of Kaplan–Meier survival indicated that GC patients with high expression of RHOT1 experienced short survival. (e) Correlations between miR-1299 and RHOT1 were confirmed at the level of GC tissue. (f) Correlations between hsa_circ_0005230 and RHOT1 were confirmed at the level of GC tissue. (g) The dual-luciferase reporter assay was conducted to verify the binding target relation between RHOT1 and miR-1299. (h) After silencing hsa_circ_0005230, the expression of RHOT1 was decreased in AGS and HGC-27 cells. (I) RHOT1 protein was up-regulated in 48 cases of GC tissues by WB. (j) Immunohistochemical staining showed RHOT1 proteins were localized to cell cytoplasm and have a positive expression in GC cells. (a. normal gastric mucosa; b. poorly differentiated adenocarcinoma without lymph node metastasis; c. The poorly differentiated adenocarcinoma with lymph node metastasized; d. mucinous adenocarcinoma). The data were expressed as mean ± SD; *P < 0.05, **P < 0.01.

## Discussion

In recent years, published research for circRNAs was mainly on aspects of its expression patterns, regulations, classic functions as miRNAs sponge, and new functions for translating protein [29–31]. Because of the wide use of high-throughput sequencing and deep development of microarray assays, numerous circRNAs are identified as aberrant expressions from various types of tumor tissues, including in hepatocellular carcinoma [32], colorectal cancer [33], lung cancer [34], and breast cancer [35]. With a covalently closed-loop structure, difficult to degrade by RNase, tissue-specific expression, and formatted large complexes of RNA-protein to exert their functions from which properties indicated that circRNAs have potential capabilities as important biomarkers to diagnose cancers and assess prognosis. CircHIPK3 was a remarkable down-regulation in bladder cancer tissues which had negatively related to cancer grades, lymph node metastasis, and invasion [36]. CircPVT1 was up-regulated in non-small cell lung cancer (NSCLC), in which patients not only had positively correlated with clinicopathological features but also worse prognosis [37]. Circ-0000190 was down-regulation in plasma and tissues of GC patients, in which expression correlated with common clinicopathological features [38]. In our study, we first provided evidence that hsa_circ_0005230 expression was up-regulated in GC tissues and cells, then using the correlation assessed the high expression of hsa_circ_0005230 and histological grade, lymphoid node metastases, and advanced TNM staging had positively correlated. Therefore, we fairly believed that hsa_circ_0005230 has a potential ability to serve as a biomarker for early diagnosis and prognosis assessment of GC, basis for personalized precision treatment.

Several studies showed that circRNAs mainly exert on cell proliferation, invasion, and migration with a kind of important regulators of various biological processes of cancers [39,40]. Aberrant expression of circRNAs correlated with the malignant biological behavior of GC cells. Huang [41] reported that circ_0008035 was up-regulated expression in GC, and silencing circ_0008035 could inhabit growth proliferation, invasion, and metastasis of GC cells. Silencing circ-PRMT5 expression could reduce the clonal formation of GC cells, increase apoptosis of GC cells [42]. To verify the aberrant expression of hsa_circ_0005230 has a positive effect on influencing the biological behavior of GC, we used silencing hsa_circ_0005230 expression to detect the changing capacities of GC cells. We found that silencing hsa_circ_0005230 significantly reduced cell viability, effectively inhibiting the clonal growth and proliferation of GC cells, besides arresting the G0/G1 phase of the cell cycle affecting cell division and proliferative activity. Furthermore, silencing hsa_circ_0005230 inhibited the capacities of invasion and migration of GC cells. The above results uncovered that hsa_circ_0005230 could have a positive effect on malignant biological behavior of GC cells.

CircRNAs using ceRNA mechanism that could act as a sponge for miRNA to regulate the expression of downstream target mRNA [24]. CircFBXL5 could compete for sponge bind to miR-660 that induces increased expression of SRSF6 [4]. Circ-0008035 sponge miR-599 to improve the expression of EIF4A1 advanced the progression of colorectal cancer [43]. In our study, we found that miR-1299 was down-regulation in GC. It had a worse prognosis in GC patients with miR-1299 down-regulation. Besides, previous studies verified that miR-1299 had specific binding sites sponging with hsa_circ_0005230 by luciferase reporter assay [14], we deeply researched on hsa_circ_0005230 could sponge miR-1299 in GC cells, and when decreased expression of hsa_circ_0005230, the expression of miR-1299 was increased meanwhile. RHOT1 as an oncogene regulated the proliferation and migration of pancreas cancer cells via SMAD4-dependent TGF-β signaling [17]. Myc transcriptionally controls a gene network of mitochondrial transport which network included RHOT1 [44]. Interfering with RHOT1 inhibited mitochondrial dynamics, which prevented the recruitment of mitochondria to the cortical cytoskeleton of tumor cells and the results have blocked the capacities of cell invasion and migration. In our study, we tested that RHOT1 was up-regulated in tissues and cells of GC, which was a high expression in GC tissue with a worse prognosis in GC patients. When silencing hsa_circ_0005230, we tested the expression of RHOT1 with decreased, also the expression of miR-1299 increased meanwhile. Additionally, miR-1299 had a special site binding to RHOT1 indicated by luciferase reporter assay. The correlations of hsa_circ_0005230, miR-1299, and RHOT1 were consistent with the predicted correlations not only at the GC cell level but also at the GC tissue level. So, according to the regulated change relationship, the hsa_circ_0005230/miR-1299/RHOT1 axis succeeded in the establishment.

EMT is one of the key steps in the process of distant metastasis of tumors. E-cadherin has an important action in maintaining the steady state of the polar epithelial monolayer in epithelial cells. During the EMT process, GC cells have an increased expression of mesenchymal markers N-cadherin, Snail and Vimentin, meanwhile a decreased expression of E-cadherin. Up-regulation of hsa_circ_0023642 expression enhances cell invasion and metastasis by regulating the EMT phenotype in GC [45]. CircNRIP1 sponged miRNA-149-5p to promote GC progression by the AKT1/mTOR pathway that exerts a positive effect on EMT to promote tumor metastasis [46]. We tested the expression of RHOT1 protein was up-regulated in GC tissue which has a positive relationship with lymphoid node metastases and TNM staging. Jiang [16] and Li [17] presented that RHOT1 affected the biological behavior of pancreatic cancer by EMT phenotype. According to the up-regulated expression of RHOT1 having a positive relationship with clinicopathological features of invasion and migration, we also speculated RHOT1 may play a role in the biological behavior of GC by EMT. In our study, we detected the changing trends of the expression of EMT phenotype main marker proteins after silencing hsa_circ_0005230 in GC cells. We verified that when hsa_circ_0005230 expression was reduced, the proliferation, invasion, and migration of GC cells were diminished, while the EMT phenotype transformed from mesenchymal to epithelialization by cellular experiments. Now that we reasonably supposed that the hsa_circ_0005230/miR-1299/RHOT1 axis could influence invasion and migration of GC by the EMT phenotype. Consequently, we could trust that hsa_circ_0005230 probably promoted increasing RHOT1 expression by binding to miR-1299 and increasing invasion and migration by the EMT mechanism in GC cells.

## Conclusion

Hsa_circ_0005230 is up-regulated expression in GC tissues and cells. It could regulate the miR-1299/RHOT1 axis, then through the EMT phenotypic pathway to influence the proliferation, invasion, and migration behavior of GC cells . The above suggested that hsa_circ_0005230/miR-1299/RHOT1 axis was successfully established not only to provide a theoretical basis for early diagnosis and prognosis assessment of GC but also expected to be new targets for the treatment of GC patients and provide theoretical support for the precision treatment of GC.Acknowledgments

None.

## Supplementary Material

Supplemental Material
