# Supplementary material for: Hsa_circ_0005230 is up-regulated and promotes gastric cancer cell invasion and migration via regulating the miR-1299/RHOT1 axis
Source: Bioengineered. 2022 Feb 16;13(3):5046–63. doi: 10.1080/21655979.2022.2036514 (PMC8973856; doi:10.1080/21655979.2022.2036514)
Supplement: Supplemental Material [file KBIE_A_2036514_SM0078.zip › supplementary/Supplementary Table 2.docx]

**Table 3 Relationship between different RHOT1 expression and clinicopathological features of GC**

| **Clinic characteristics** | **Total** | **RHOT1** | | **χ^2^** | ***P*-value** |
| --- | --- | --- | --- | --- | --- |
|  |  | **High(%)** | **Low** |  |  |
| **Gender** |  |  |  |  | 0.168 |
| Male | 41 | 30(73.2) | 11 |  |  |
| Female | 10 | 5(50) | 5 |  |  |
| **Age(year)** |  |  |  | 0.219 | 0.64 |
| ≤60 | 15 | 11(73.3) | 4 |  |  |
| ＞60 | 36 | 24(66.7) | 12 |  |  |
| **Location** |  |  |  |  | 0.67 |
| Gastroesophageal | 4 | 3(75) | 1 |  |  |
| Fundus/Cardia | 2 | 1(50) | 1 |  |  |
| body | 12 | 9(75) | 3 |  |  |
| Antrum | 31 | 20(64.5) | 11 |  |  |
| Total stomach | 2 | 2(100) | 0 |  |  |
| **Tumor size(cm)** |  |  |  | 0.081 | 0.776 |
| ≤5 | 24 | 16(66.7) | 8 |  |  |
| ＞5 | 27 | 19(70.4) | 8 |  |  |
| **Gross types** |  |  |  |  |  |
| EGC |  |  |  |  |  |
| I | 1 | 0(0) | 1 |  |  |
| III | 1 | 0(0) | 1 |  |  |
| AGC |  |  |  |  | 0.24 |
| Bor.I+II | 2 | 2(100) | 0 |  |  |
| Bor.III+IV | 47 | 33(70.2) | 14 |  |  |
| **WHO’s histological types** |  |  |  |  | 0.663 |
| Papillary adenocarcinoma | 3 | 2(66.7) | 1 |  |  |
| Tubular adenocarcinoma |  |  |  |  |  |
| Well differentiated | 2 | 1(50) | 1 |  |  |
| Moderately differentiated | 15 | 11(73.3) | 4 |  |  |
| Poorly differentiated adenocarcinoma | 18 | 14(77.8) | 4 |  |  |
| Undifferentiated carcinoma | 1 | 1(100) | 0 |  |  |
| Signet ring cell carcinoma | 7 | 4(57.1) | 3 |  |  |
| Mucinous adenocarcinoma | 5 | 2(40) | 3 |  |  |
| **Histological grade** |  |  |  |  | 0.716 |
| G1 | 4 | 2(50) | 2 |  |  |
| G2 | 14 | 10(71.4) | 4 |  |  |
| G3 | 33 | 23(69.7) | 10 |  |  |
| **Lauren’s types** |  |  |  |  | 0.859 |
| Intestinal | 12 | 9(75) | 3 |  |  |
| Diffuse | 30 | 20(66.7) | 10 |  |  |
| Mixed | 9 | 6(66.7) | 3 |  |  |
| **Depth of invasion(T)** |  |  |  |  | 0.195 |
| T1+T2 | 3 | 1(33.3) | 2 |  |  |
| T3+T4 | 48 | 34(70.8) | 14 |  |  |
| **Lymph node metastasis (N)** |  |  |  |  | **0.035*** |
| N0 | 11 | 5(45.5) | 6 |  |  |
| N1-2 | 18 | 11(61.1) | 7 |  |  |
| N3 | 22 | 19(86.4) | 3 |  |  |
| **Distant metastasis (M)** |  |  |  |  | 0.576 |
| M0 | 49 | 34(69.4) | 15 |  |  |
| M1 | 2 | 1(50) | 1 |  |  |
| **TNM staging** |  |  |  |  | **0.042*** |
| I | 2 | 0(0) | 2 |  |  |
| II | 12 | 6 (50) | 6 |  |  |
| III | 33 | 26(78.8) | 7 |  |  |
| IV | 4 | 3(75) | 1 |  |  |

Note: *P<0.05.

Abbreviation: TNM, tumor–node–metastasis.

EGC, early gastric carcinoma

AGC, advanced gastric carcinoma
