# Supplementary material for: Hsa_circ_0005230 is up-regulated and promotes gastric cancer cell invasion and migration via regulating the miR-1299/RHOT1 axis
Source: Bioengineered. 2022 Feb 16;13(3):5046–63. doi: 10.1080/21655979.2022.2036514 (PMC8973856; doi:10.1080/21655979.2022.2036514)
Supplement: Supplemental Material [file KBIE_A_2036514_SM0078.zip › supplementary/Supplementary Table 3.docx]

**Supplementary Table 3 Immunochemical staining of RHOT1 expressions**

| Items | Total | RHOT1 expression | | X value | *P-*value | |
| --- | --- | --- | --- | --- | --- | --- |
|  |  | High（%） | Low |  |  |  |
| Gastric cancer | 166 | 63(40.6) | 92 | 67.254 | <0.01 | |
| Normal gastric mucosa | 136 | 1(0.7) | 135 |  |  |  |
