# Supplementary material for: Hsa_circ_0005230 is up-regulated and promotes gastric cancer cell invasion and migration via regulating the miR-1299/RHOT1 axis
Source: Bioengineered. 2022 Feb 16;13(3):5046–63. doi: 10.1080/21655979.2022.2036514 (PMC8973856; doi:10.1080/21655979.2022.2036514)
Supplement: Supplemental Material [file KBIE_A_2036514_SM0078.zip › supplementary/Supplementary Table 4.docx]

**Table 6** **Relationship between GC immunohistochemical staining for RHOT1 differential expression and clinicopathological features.**

| **Clinic characteristics** | **Total** | **hsa_circ_0005230** | | **χ^2^** | ***P*-value** |
| --- | --- | --- | --- | --- | --- |
|  |  | **High(%)** | **Low** |  |  |
| **Gender** |  |  |  |  |  |
| Male | 112 | 43(38.4) | 69 | 0.849 | 0.357 |
| Female | 43 | 20(46.5) | 23 |  |  |
| **Age(year)** |  |  |  | 0.118 | 0.731 |
| ≤60 | 69 | 27(39.1) | 42 |  |  |
| ＞60 | 86 | 36(41.9) | 50 |  |  |
| **Location** |  |  |  |  | 0.244 |
| Gastroesophageal | 6 | 5(83.3) | 1 |  |  |
| Fundus/Cardia | 10 | 3(30) | 7 |  |  |
| body | 41 | 15(36.6) | 26 |  |  |
| Antrum | 90 | 37(41.1) | 53 |  |  |
| Total stomach | 8 | 3(37.5) | 5 |  |  |
| **Tumor size(cm)** |  |  |  | 0.009 | 0.923 |
| ≤5 | 77 | 31(40.3) | 46 |  |  |
| ＞5 | 78 | 32(41) | 46 |  |  |
| **General types** |  |  |  |  | 0.587 |
| Bor.I+II | 12 | 4(33.3) | 8 |  |  |
| Bor.III+IV | 143 | 59 | 84 |  |  |
| **WHO’s histological types** |  |  |  | 8.347 | 0.054 |
| Papillary adenocarcinoma | 5 | 0(0) | 5 |  |  |
| Tubular adenocarcinoma |  |  |  |  |  |
| Moderately differentiated | 35 | 11(31.4) | 24 |  |  |
| Poorly differentiated adenocarcinoma | 105 | 47(44.8) | 58 |  |  |
| Undifferentiated carcinoma | 1 | 1(100) | 0 |  |  |
| Signet ring cell carcinoma | 1 | 1(100) | 0 |  |  |
| Mucinous adenocarcinoma | 8 | 3(37.5) | 5 |  |  |
| **Histological grade** |  |  |  |  | **0.03** |
| G1 | 5 | 0(0) | 5 |  |  |
| G2 | 34 | 11(32.4) | 23 |  |  |
| G3 | 116 | 52(44.8) | 64 |  |  |
| **Lauren’s types** |  |  |  |  | **0.012** |
| Intestinal | 36 | 7(19.4) | 29 |  |  |
| Diffuse | 38 | 17(44.7) | 21 |  |  |
| Mixed | 81 | 39(48.1) | 42 |  |  |
| **Depth of invasion(T)** |  |  |  |  | **0.038** |
| T2 | 12 | 3(25) | 9 |  |  |
| T3 | 43 | 12(27.9) | 31 |  |  |
| T4 | 100 | 48(48) | 52 |  |  |
| **Lymph node metastasis (N)** |  |  |  | 5.929 | **0.015** |
| N0 | 35 | 8(22.9) | 27 |  |  |
| N1-3 | 120 | 55(45.8) | 65 |  |  |
| **TNM staging** |  |  |  | 14.74 | **<0.01** |
| I+II | 49 | 9(18.4) | 40 |  |  |
| III+IV | 106 | 54(50.9) | 52 |  |  |

Note: *P<0.05.

Abbreviation: TNM, tumor–node–metastasis.
