# Supplementary figures and images for: Hsa_circ_0005230 is up-regulated and promotes gastric cancer cell invasion and migration via regulating the miR-1299/RHOT1 axis
Source: Bioengineered. 2022 Feb 16;13(3):5046–63. doi: 10.1080/21655979.2022.2036514 (PMC8973856; doi:10.1080/21655979.2022.2036514)

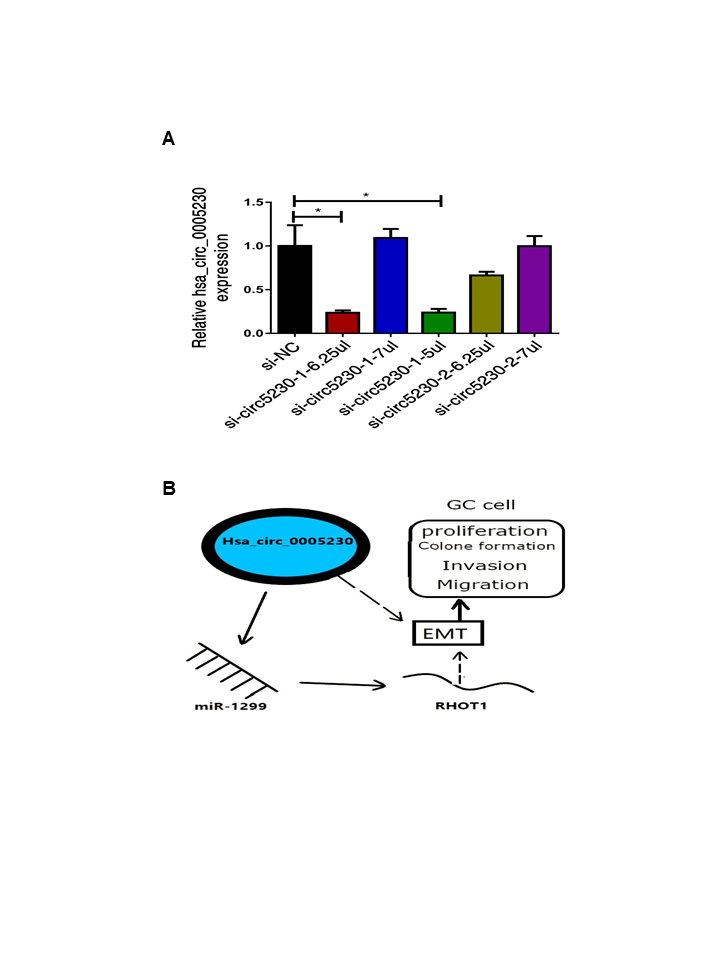

Supplement: Supplemental Material [file KBIE_A_2036514_SM0078.zip › supplementary/Figure supplementary.tif]
